# Presurgical epilepsy evaluation and epilepsy surgery

**DOI:** 10.12688/f1000research.17714.1

**Published:** 2019-10-29

**Authors:** Christoph Baumgartner, Johannes P. Koren, Martha Britto-Arias, Lea Zoche, Susanne Pirker

**Affiliations:** 1 Department of Neurology, General Hospital Hietzing with Neurological Center Rosenhügel, Vienna, Austria; 2 Karl Landsteiner Institute for Clinical Epilepsy Research and Cognitive Neurology, Vienna, Austria; 3 Medical Faculty, Sigmund Freud University, Vienna, Austria

**Keywords:** epilepsy, surgery, presurgical evaluation

## Abstract

With a prevalence of 0.8 to 1.2%, epilepsy represents one of the most frequent chronic neurological disorders; 30 to 40% of patients suffer from drug-resistant epilepsy (that is, seizures cannot be controlled adequately with antiepileptic drugs). Epilepsy surgery represents a valuable treatment option for 10 to 50% of these patients. Epilepsy surgery aims to control seizures by resection of the epileptogenic tissue while avoiding neuropsychological and other neurological deficits by sparing essential brain areas. The most common histopathological findings in epilepsy surgery specimens are hippocampal sclerosis in adults and focal cortical dysplasia in children. Whereas presurgical evaluations and surgeries in patients with mesial temporal sclerosis and benign tumors recently decreased in most centers, non-lesional patients, patients requiring intracranial recordings, and neocortical resections increased. Recent developments in neurophysiological techniques (high-density electroencephalography [EEG], magnetoencephalography, electrical and magnetic source imaging, EEG-functional magnetic resonance imaging [EEG-fMRI], and recording of pathological high-frequency oscillations), structural magnetic resonance imaging (MRI) (ultra-high-field imaging at 7 Tesla, novel imaging acquisition protocols, and advanced image analysis [post-processing] techniques), functional imaging (positron emission tomography and single-photon emission computed tomography co-registered to MRI), and fMRI significantly improved non-invasive presurgical evaluation and have opened the option of epilepsy surgery to patients previously not considered surgical candidates. Technical improvements of resective surgery techniques facilitate successful and safe operations in highly delicate brain areas like the perisylvian area in operculoinsular epilepsy. Novel less-invasive surgical techniques include stereotactic radiosurgery, MR-guided laser interstitial thermal therapy, and stereotactic intracerebral EEG-guided radiofrequency thermocoagulation.

## Introduction

Epilepsy has a prevalence of 0.8 to 1.2% and thus represents one of the most frequent chronic neurological disorders ^1, 2^. In about 30 to 40% of patients, seizures cannot be controlled adequately with antiepileptic drugs; that is, these patients suffer from drug-resistant epilepsy ^3, 4^. Epilepsy surgery represents a valuable treatment option for 10 to 50% of these patients ^4^ and has been shown to be significantly superior to continued antiepileptic drug treatment regarding seizure control and quality of life in both children and adults ^5– 7^. The goals of epilepsy surgery are seizure control by resection of the epileptogenic tissue on the one hand while sparing essential brain areas to avoid neuropsychological and other neurological deficits on the other hand ^3, 4, 8– 10^. This can be achieved only by a thorough presurgical evaluation clearly delineating epileptogenic and essential brain areas and by defining selective resection strategies in each individual patient ^3, 8– 10^. Several excellent articles recently reviewed presurgical evaluation and surgical treatment of patients with drug-resistant epilepsy ^3, 4, 8– 10^. Here, we will focus on some selected recent developments, but we make no claim of providing an exhaustive review.

## Trends in presurgical evaluation and surgical treatment in the last few decades

Blumcke *et al*. ^11^ reported the largest series of histopathological findings of resected brain tissue. The tissue in this series was obtained from 9523 patients who underwent epilepsy surgery for drug-resistant epilepsy in 36 European centers between 1990 and 2014. The most common pathologies were hippocampal sclerosis (36.4%), tumors (mainly ganglioglioma; 23.6%), and malformations of cortical development (19.8%). No histopathological diagnosis could be established in 7.7%. Whereas the most common diagnosis in adults was hippocampal sclerosis (44.5%), focal cortical dysplasia represented the most prevalent finding in children (39.3%). Several significant changes in patients undergoing presurgical evaluation and epilepsy surgery occurred in the last few decades ^12– 18^. Whereas presurgical evaluations continually increased over time, surgical interventions remained stable or decreased in recent years, resulting in an increasing number of evaluated patients ultimately not undergoing surgery ^18, 19^. Specifically, the number of patients with mesial temporal sclerosis and benign tumors who were evaluated and ultimately underwent surgery decreased over time in most centers. The same holds true for the number of temporal lobe resections in general ^16, 18^. Reasons for the decrease of mesial temporal epilepsy surgery are intensively debated and could be explained by a decreasing incidence of Ammon’s horn sclerosis, by a reduction of the prevalent pool, or by alternative treatment options available for these patients ^15, 19– 21^. On the contrary, non-lesional patients, patients requiring intracranial recordings, and neocortical resections increased ^16, 18^. Furthermore, more evaluated patients did not undergo surgery since patients were not suitable for surgery (a circumstance due mainly to missing identification of a circumscribed epileptogenic zone) or since more patients offered surgery by physicians opted against it ^18^. Finally, in some centers, an increase in patients willing to undergo less-invasive surgeries—for example, stereotactic radiosurgery, magnetic resonance (MR)-guided laser interstitial thermal therapy, stereotactic intracerebral electroencephalography (SEEG)-guided radiofrequency thermocoagulation (RFTC), deep brain stimulation (DBS), and responsive neurostimulation (RNS)—who had previously refused open surgery can be observed ^22^.

## Presurgical evaluation – general issues

Each presurgical evaluation starts with a so-called phase 1 evaluation. Investigations required in all patients include high-resolution MR imaging (MRI), video scalp electroencephalography (EEG), and detailed neuropsychological assessment ^8, 23^.

If these investigations yield converging evidence on the localization of the epileptogenic zone and provide sufficient information on the risk of potential postoperative deficits, the patient may be referred to surgery directly.

If there are ambiguous results concerning localization of the epileptogenic zone, the following additional phase 1 investigations can be applied ^8, 24^:

- advanced structural imaging techniques for detection of epileptogenic lesions on MRI;

- interictal high-resolution EEG (HD-EEG), interictal magnetoencephalography (MEG), interictal electrical source imaging (ESI), interictal magnetic source imaging (MSI), and interictal EEG-functional MRI (EEG-fMRI) for accurate delineation of the irritative zone ^25^;

- interictal 18F-fluoro-deoxyglucose (FDG) positron emission tomography (PET) or interictal PET with other tracers to localize the functional deficit zone;

- ictal single-photon emission computed tomography (SPECT), ictal HD-EEG, ictal ESI, and in selected cases ictal MSI and ictal EEG-fMRI to localize the ictal onset zone.

Depending on the lateralization and localization of the planned resection, the risk of potential postoperative deficits can be further clarified by the following complementary phase 1 investigations ^8^:

- fMRI, MEG, and Wada test for lateralization and localization of language functions;

- fMRI and Wada test for prediction of postoperative memory decline;

- tractography of Meyer’s loop and visual field testing to assess the risk of postoperative visual field defects;

- fMRI and tractography of pyramidal tract to assess the risk of postoperative motor deficits.

Although most centers agree on the essential methods of phase 1 evaluation (that is, high-resolution MRI, video scalp EEG, and detailed neuropsychological assessment), there is a lot of variability across the international community, and some routinely require procedures (for example, PET, SPECT, and MEG) that others may relegate to later stages of the evaluation. If the epileptogenic zone cannot be localized with sufficient certainty with these non-invasive techniques, if non-invasive techniques yield divergent results and/or if the presumed epileptogenic zone is located in close proximity to functionally important brain areas and the patient is still considered a reasonable surgical candidate, intracranial EEG (IEEG) can be applied during a so-called phase 2 evaluation ^8, 24– 26^.

Chronic extraoperative IEEG techniques include the following:

- extraoperative IEEG through open craniotomy (CEEG); after the open craniotomy, subdural grids or strips or a combination of depth electrodes and subdural electrodes are placed under direct observation;

- SEEG using intracerebral depth electrodes; usually 5–18 multicontact electrodes are inserted through a twist drill hole or burr hole under general anesthesia. The electrodes are placed stereotactically with a frame, under neuronavigational guidance, or, robotic assistance. The trajectories of the electrodes have to be planned thoroughly to avoid crossing blood vessels;

- hybrid extraoperative EEG (HEEG) consisting of a hybrid of CEEG and SEEG;

- foramen ovale IEEG with multicontact electrodes placed to lie along the long axis of the hippocampus (usually bilaterally);

- epidural IEEG using epidural peg electrodes, allowing sampling from several cortical areas, even distant ones ^26^.

It should be mentioned that in many centers there has been a shift from CEEG to SEEG, which is less invasive and much more comfortable for patients. Ideally, the modality best suited for each individual patient should be applied in accordance with standardized protocols ^26^.

Intraoperative electrocorticography (ECoG) where IEEG recordings and electrical stimulation mapping (ESM) are performed intraoperatively prior to, during, and often following resection using subdural, depth, or wick electrodes placed under direct visualization or guided by neuronavigation represents an additional option, especially in patients with cortical dysplasia, tuberous sclerosis, or scalp EEG with continuous epileptiform discharges (CEDs), when extraoperative intracranial EEG is not feasible and finally when surgery is performed adjacent to language or motor cortex ^25^. The diagnostic utility, strengths and limitations, risks/morbidities, and specific indications of various IEEG techniques were summarized in a consensus-based expert recommendation ^26^.

## Neurophysiological techniques

Recent advances in clinical neurophysiology include localization of the irritative zone (that is, the interictal spike zone with ESI, MSI, and EEG-fMRI). ESI and MSI represent biophysical models which allow us to calculate from the EEG resp. MEG signals measured on the scalp the intracerebral neuronal sources generating these signals, that is solving the so-called inverse problem of clinical neurophysiology ^27, 28^. Localization accuracy and sensitivity of EEG can be significantly improved when the number of electrodes is increased beyond the standard 10-20 system ^29^ and when montages sampling inferior temporal areas with electrodes around and below the ears and on the cheeks and neck are used ^30, 31^. HD-EEG set-ups currently use 128 to 256 EEG electrodes ^32, 33^. In a study comparing various non-invasive techniques (that is, structural MRI, HD-ESI, PET, and SPECT), the combination of MRI and HD-ESI offered the highest predictive value for postoperative seizure freedom ^34^. The sensitivity of MEG for the detection of interictal spikes on ECoG was assessed in a study using simultaneous MEG and ECoG recordings ^35^. Of all of the interictal ECoG spikes, 56% had an interictal MEG counterpart. The rates of association between MEG and ECoG were at least 90% in the interhemispheric and frontal orbital regions; about 75% in the superior frontal, central, and lateral temporal regions; but only about 25% in the mesial temporal region ^35^. Recently, spectral and functional connectivity properties of non-invasively obtained HD-EEG and MEG virtual electrodes matched those of SEEG recordings. HD-EEG and MEG virtual electrodes therefore might be used for optimal SEEG planning, making the localization of the seizure onset zone safer and more successful ^36^. The yield of simultaneous scalp EEG-fMRI studies (40 to 70% of recordings remain inconclusive and this is due principally to the absence of interictal epileptiform discharges during simultaneous recordings or lack of hemodynamic changes correlated to interictal epileptiform discharges) can be significantly increased by building epilepsy-specific electroencephalographic voltage maps using averaged interictal epileptiform discharges recorded during long-term clinical monitoring outside the scanner, computing the correlation of these maps with EEG recordings in the scanner for each time frame, and using the time course of this correlation coefficient as a regressor for fMRI analysis to map hemodynamic changes related to these epilepsy-specific maps (topography-related hemodynamic changes) ^37^. With this technique, scalp EEG-fMRI results can be obtained even in the absence of visually detectable spikes during the EEG-fMRI session ^37^. In a recent study in 53 children including MRI-negative cases, the combination of EEG-fMRI with ESI could localize the seizure onset zone with high accuracy and predicted surgical outcome in all patients ^38^.

Localizations obtained by interictal ESI, MSI, and EEG-fMRI showed excellent agreement both with the irritative zone delineated by intracranial electrodes and with the epileptogenic zone defined by other investigations ^32, 35, 36, 39, 40^. Furthermore, complete resection of the irritative zone defined by these methods resulted in a favorable surgical outcome ^32, 38, 39, 41^.

Recently, ictal ESI was successfully applied for localization of the ictal onset zone ^42– 50^. Ictal MSI is also feasible but is limited by the fact that long-term MEG recordings cannot be performed and it remains difficult to catch seizures during short-term MEG recordings ^51– 54^.

It should be noted that automated techniques exist to perform both interictal and ictal ESI ^25, 42^.

Pathological high-frequency oscillations (pHFOs) in the frequency range of 100 to 600 Hz are considered biomarkers of epileptogenic tissue ^55– 58^. It could be shown that pHFOs, while initially recorded only on invasive EEG, can also be recorded non-invasively on scalp EEG and MEG ^57, 59– 62^. pHFOs are of high localizing significance, and removal of brain areas generating pHFOs predicts a favorable postsurgical seizure control ^57, 63– 65^. Recently, it was shown that pHFO activity may change during surgery and that removal of post-resection pHFO can further improve surgical outcome ^66^. However, the added value of pHFOs compared with interictal spikes is still discussed in the community and combined measures of spikes and pHFOs might improve identification of epileptogenic tissue ^67^.

Cortico-cortical evoked potentials (CCEPs) in conjunction with neuroimaging techniques like diffusion tensor imaging (DTI) have recently been successfully applied to identify both epileptogenic and functional networks ^68– 72^.

## Structural neuroimaging

Progress in structural neuroimaging has revolutionized presurgical epilepsy evaluation in recent years ^73– 75^. Structural neuroimaging aims to identify an epileptogenic lesion responsible for the patient’s seizures which in turn significantly increases the chances of postoperative seizure freedom ^76, 77^. However, 15 to 30% of patients with drug-resistant epilepsy remain MRI-negative; that is, no structural lesion can be identified ^73, 78, 79^. A widely accepted imaging protocol for epilepsy-specific imaging based on six sequences could identify 99.4% of 2740 epileptogenic lesions and provides a reasonable balance between diagnostic accuracy and clinical feasibility ^80^. This protocol includes 3D volumetric T1-weighted imaging (1-mm isotropic voxels) (detection of malformations of cortical development and application of post-processing techniques), axial and coronal T2-weighted (T2/short tau inversion recovery [STIR]) imaging (assessment of hippocampal architecture and cystic tissue components of other lesions), axial and coronal fluid-attenuated inversion recovery (FLAIR) sequences (detection of hippocampal sclerosis, focal cortical dysplasia, tumors, inflammation, and scars), and axial T2* gradient echo or susceptibility-weighted (Hemo/Calc) sequences (identification of vascular and calcified lesions such as cavernomas and arteriovenous malformations) ^73, 80^. 

The detection of lesions, especially of focal cortical dysplasia and hippocampal sclerosis, can be significantly increased by improvements of imaging hardware (including ultra-high-field imaging at 7 Tesla), by novel imaging acquisition protocols, and by advanced image analysis (post-processing) techniques ^73^.

Ultra-high-field imaging at 7-Tesla MRI in patients with hippocampal sclerosis showed a strong correlation between MRI and histology with sensitivity and specificity values up to 100% for the detection of pathology in various hippocampal subfields ^81^. Seven-Tesla MRI including whole-brain FLAIR and gradient-recalled echo (GRE) images could detect epileptogenic focal cortical dysplasia not visible at conventional field strengths in a third of cases, while gliosis remained undetected ^82^.

Advances in imaging acquisition protocols can be useful to detect lesions which cannot be identified by using routine imaging protocols ^73^. Double inversion recovery suppresses the signals from both cerebrospinal fluid (CSF) and normal white matter and therefore enhances the detection of cortical lesions otherwise masked by white matter or CSF signal ^83^. Arterial spin labeling provides a quantitative measure of regional cerebral blood flow (rCBF) and demonstrated a reduced regional perfusion in the seizure onset zone - in good agreement with hypometabolism on PET - in MRI-negative patients ^84^. Neurite orientation dispersion and density imaging (NODDI) represents an advanced diffusion imaging technique that provides more detailed information on tissue microstructure, including intracellular volume fraction, a marker of neurite density, and was helpful to identify focal cortical dysplasia in MRI-negative patients ^85^.

Several post-processing methods, including voxel-based morphometry, T2-relaxometry, surface-based morphometry, or DTI recently combined with machine learning approaches, can be useful for the identification of lesions not apparent on visual MRI analysis ^73, 74, 86– 94^.

## Neuropsychological assessment

Cognitive impairments in epilepsy can be caused by underlying pathology, seizures, interictal spikes, antiepileptic drugs, and psychiatric comorbidities ^95^. Although neuropsychological assessment remains an essential investigation during presurgical evaluation, the advent of high-resolution structural and functional brain imaging as well as sophisticated EEG analysis techniques has shifted the main focus of neuropsychology from localization toward prediction of postsurgical cognitive outcome and quality control of epilepsy surgery ^95– 97^. The major factors determining postsurgical neuropsychological performance include functional integrity of the resected tissue, functional reserve capacity of the remaining brain, the degree of functional plasticity, postoperative control of epileptic activity, and the effects of quantitative and qualitative postsurgical antiepileptic drug changes ^10, 97– 99^. However, it is still not possible to predict with certainty which patient will experience disabling functional loss on an individual scale ^100^. Several studies showed stable or even improved long-term postsurgical cognitive outcomes, especially in seizure-free patients ^101– 103^. In regard to cognitive outcomes of different surgical approaches, individual and selective surgery preserving functional brain tissue and fiber tracts, thus minimizing collateral damage, should be preferred ^104^. Although radiosurgery or thermocoagulation should be advantageous from this perspective, no clear scientific evidence of a superior cognitive outcome of these procedures compared with conventional open surgery currently exists ^98^. Recommendations for a standardized neuropsychological assessment in the preoperative evaluation and postoperative follow-up of epilepsy surgery patients have been published by the International League Against Epilepsy (ILAE) Neuropsychology Task Force Diagnostic Methods Commission ^105, 106^. Neuropsychological assessments of patients undergoing MRI-guided stereotactic laser amygdalohippocampectomy resulting in very small focal lesions showed outcomes not consistent with prevailing models of brain function. Thus, the hippocampus does not appear to be an essential component of neural networks underlying naming, verbal fluency, object and person recognition, and declarative verbal memory ^107, 108^. Furthermore, white matter tracts have been proven to be crucial for language and semantic networks. The basal temporal language area is likely a critical hub in the language network ^109^. Therefore, MRI-guided stereotactic laser amygdalohippocampectomy offers a unique opportunity to study structure–function brain relationships and to reappraise many of our models of cognitive networks ^107^. Moreover, traditional neuropsychological assessment does not include many cognitive domains of potentially high clinical relevance, including category-specific naming and fluency as well as object and face recognition, for epilepsy patients in their everyday lives ^110^. Future neuropsychological testing should integrate sensory and linguistic/semantic information with the existing knowledge of the patient, explore memory consolidation over long periods of time, and attempt to relate cognition to features of signal processing in order to better assess cognitive networks and to provide objective measures accounting for subjective cognitive complaints of the patients ^110^.

## Positron emission tomography and single-photon emission computed tomography

The role of PET and SPECT in presurgical evaluation was recently reviewed by several authors ^8, 73, 74, 111^. FDG-PET assessing interictal brain dysfunction (that is, the functional deficit zone) has been used during presurgical evaluation for more than 35 years ^112^. In mesial temporal lobe epilepsy (MTLE), FDG-PET shows a widespread ipsilateral hypometabolism involving the temporal lobe (that is, mesial temporal structures, temporal pole, and lateral temporal cortex) as well as extratemporal areas (including the insula, the frontal lobe, perisylvian regions, and the thalamus). This hypometabolism is more pronounced in right as compared with left MTLE ^113^. The topography of hypometabolism on PET showed a strong correlation with the extent of the electroclinical network defined by clinical seizure semiology and EEG ^113^. Homotopic contralateral hypermetabolism indicating possible compensatory mechanisms was relatively higher in patients with left-sided MTLE and in female patients but was lower with longer disease duration, later onset of epilepsy, and higher seizure frequency ^113^. These findings indicate that MTLE is not a focal but rather a network disease that can affect interconnected and even distant brain areas ^114, 115^. Hypometabolic patterns on FDG-PET were predictive for surgical outcome in patients with MTLE ^116, 117^. Specifically, non-class IA outcome correlated with extratemporal metabolic changes whereas class IA outcome was associated with a focal anteromesial temporal hypometabolism ^116^.

In MRI-negative temporal lobe epilepsy (TLE), a hypometabolism ipsilateral to the presumed epileptogenic zone is a predictor for a favorable surgical outcome ^118^. Thus, in patients with MRI-negative FDG-PET–positive TLE, an excellent surgical outcome can be achieved after anterior temporal lobectomy (and this is very similar to the outcome in patients with hippocampal sclerosis on MRI) ^119– 121^. These patients probably do not need to undergo intracranial recordings, especially if seizures arise from the non-dominant temporal lobe ^120, 121^.

In MRI-negative extratemporal lobe epilepsy (ETLE), FDG-PET co-registered with MRI is highly sensitive to detect focal cortical dysplasia and thus significantly improves diagnosis and surgical outcome of these patients ^122^. In a recent series of patients with histologically proven focal cortical dysplasia type 2 with negative or doubtful MRI, FDG-PET co-registered with MRI correctly localized the focal cortical dysplasia in 83% of patients, resulting in excellent surgical outcome after limited resections (seizure-free outcome in 94% of patients and Engel class IA in 72%) ^123^. Automated easy-to-use quantification of FDG PET-computed tomography was clearly superior to visual analysis for the identification of the epileptogenic zone in patients with probable frontal cortical dysplasia (concordance for automated quantification 72.7% versus 22.7% for visual analysis) ^124^. Recently, machine learning approaches using a classifier based on optimized cortical surface sampling of combined MRI and PET features were superior to both quantitative MRI and multimodal visual analysis for the detection of focal cortical dysplasia (93% versus 82% versus 68%) ^125^.

Ictal SPECT provides information on changes of rCBF—which is considered a surrogate marker of increased neuronal activity—in the seizure onset zone. Ictal SPECT is applied primarily in MRI-negative extratemporal cases or in patients with discordant findings from other investigations ^74^. The sensitivity and specificity of visual ictal SPECT interpretation (that is, visual comparison of ictal and interictal studies) can be significantly improved by subtraction SPECT co-registered to MRI (SISCOM) (that is, subtracting interictal from ictal SPECT with co-registration to MRI) ^126^. However, a major limitation of SISCOM is that normal physiologic variations between scans cannot be accounted for ^127^. Statistical parametric mapping (SPM)-based methods using a normal database can identify changes in rCBF which are statistically significantly different from normal on a voxel-by-voxel basis. One of these methods—statistical ictal SPECT co-registered to MRI (STATISCOM)—was superior to SISCOM in patients with TLE ^128^. Recently, a commercially available and easy-to-use software package (MIMneuro, MIM Software Inc., Cleveland, OH, USA) became available for ictal/interictal SPECT and MRI analysis. In a recent article, these three methods were systematically compared ^127^. STATISCOM, closely followed by MIMneuro, showed the best performance for seizure localization, and both were superior to SISCOM ^127^.

## Functional magnetic resonance imaging

fMRI is used mainly for localization of eloquent cortex and to predict postoperative language as well as memory outcomes ^8, 73, 74^. Recently, the American Academy of Neurology (AAN), in a practice guideline, assessed diagnostic accuracy and prognostic value of fMRI in determining lateralization and predicting postsurgical language and memory outcomes ^129^.

The authors performed a meta-analysis and concluded that language lateralization based on fMRI was concordant with the Wada test in MTLE (concordance rate of 87%) and in ETLE (concordance rate of 81%) but that data were insufficient for temporal tumors or lateral temporal cases ^129^. Indeed, fMRI has replaced the Wada test for language lateralization in many centers ^73, 74^. According to the practice guideline, fMRI is possibly effective to predict postsurgical language deficits in patients undergoing temporal lobectomy ^129^. A stronger left temporal activation during a semantic decision task predicted a greater postoperative naming decline with a sensitivity of 100%, a specificity of 73%, and a positive predictive value of 81% and was superior to the predictive value of the Wada test (sensitivity of 92%, specificity of 45%, and positive predictive value of 67%) ^130^. In patients with left TLE, left frontal MRI activation during a verbal fluency task predicted a postoperative decline in verbal naming after left temporal lobe resections with good sensitivity (100%) but poor specificity (33%) and a positive predictive value of 60% ^131^. Auditory and visual naming tasks eliciting anterior temporal activations are more related to naming and can provide more consistent predictive values with higher specificity ^132^. A recent machine learning study used a support vector regression (SVR) model and the results of multimodal presurgical language mapping, including fMRI, MEG, transcranial magnetic stimulation, and high-gamma ECoG (hgECoG), to predict postoperative language outcome. The SVR model consisting of fMRI and MEG was the optimal model that facilitated the best trade-off between model complexity and prediction accuracy ^133^.

In regard to language localization in patients with resections close to language cortex, cortical stimulation mapping from chronically indwelling electrodes or during awake craniotomy is generally considered the necessary gold standard ^73, 134^. However, in a recent study in patients with frontal lobe epilepsy, a significant postoperative naming decline was observed when the resection overlapped with language fMRI activation, even when electrocortical stimulation results were negative for language function in these areas ^135^. These findings support the role of fMRI for presurgical language localization ^74^.

In patients with TLE, re-organization of memory-encoding networks involves both ipsi- and contra-lateral temporal but also extratemporal brain areas ^136^. Furthermore, dynamic postoperative changes of these networks occur after left and right temporal lobe resections in both verbal and visual domains ^137^. Specifically, the contralateral hippocampus influences memory outcome 12 months after surgery ^137^. The AAN practice guideline recommended memory fMRI to lateralize and predict verbal and non-verbal memory outcome after temporal lobe surgery ^129^. In left TLE, left anterior hippocampal but also left frontal fMRI activation during verbal encoding correlated significantly with greater verbal memory decline after left anterior temporal lobe resections while ipsilateral posterior hippocampal activation was associated with better postoperative verbal memory outcome ^138, 139^. In right TLE, predominantly right anterior hippocampal activation during face encoding was predictive of a greater decline of visual memory after right anterior temporal lobe resection while predominantly right-sided posterior hippocampal activation correlated with better postoperative visual memory ^138^. In a recent study, lateralization of memory fMRI activations using a picture recognition paradigm predicted postoperative verbal and visual memory outcome independent of the type of lesion, the side of the epileptic focus, or the type of preoperative memory profile (typical or atypical) ^140^.

Nevertheless, it should be mentioned that, during presurgical epilepsy evaluation in a clinical setting, correct and clinically useful interpretation of fMRI strongly depends on the individual investigator’s and center’s expertise. Indeed, the authors of the AAN practice guideline stated that there is still a great need for further research in this area and that clinicians should carefully advise patients of the risks and benefits of fMRI versus Wada test during discussions concerning the choice of a specific modality in each individual case ^129^. In conclusion, both the Wada test and fMRI have their specific indications and limitations.

## Surgical techniques

Resective epilepsy surgery is still primarily lesion-directed because complete resection of an epileptogenic lesion represents the major determinant for a favorable surgical outcome ^8^. In non-lesional TLE, surgery can be performed on the basis of non-invasive phase 1 investigations, especially if seizures arise from the non-dominant hemisphere. On the contrary, in non-lesional extratemporal epilepsies, resections have to rely on an exact delineation of the electro-clinically defined seizure onset zone by intracranial recordings ^26, 141^.

Epilepsy surgeries requiring an operculoinsulectomy pose significant difficulties because the perisylvian area is highly vascular, deep, and functional. Recently, successful surgical treatment of operculoinsular epilepsy (Engel class I seizure control in 80% of patients) with an acceptable long-term complication rate was reported ^142^.

Novel surgical techniques in clinical use include stereotactic radiosurgery, MR-guided laser interstitial thermal therapy (MgLiTT), and SEEG-guided RFTC ^8, 10^.

A 2016 meta-analysis on stereotactic radiosurgery in TLE including 13 studies showed a pooled seizure-free rate of 50.9% (with a significant heterogeneity between studies ranging from 0 to 86%) ^143^. The most frequent adverse events were visual field deficits, headache, and verbal memory impairment ^143^. A systematic review and practice guideline of the International Stereotactic Radiosurgery Society concluded that radiosurgery was effective to control seizures in MTLE (possibly with better neuropsychological outcomes and quality-of-life metrics in selected subjects compared with microsurgery) and that radiosurgery had a better risk-benefit ratio for small hypothalamic hamartomas compared with surgical methods. However, the delayed therapeutic effect with ongoing seizures carries significant morbidity and mortality risks. For other indications, including corpus callosotomy, cavernomas malformations, and ETLE, evidence was insufficient to make recommendations ^144^.

MgLiTT entails the focused application of thermal energy in the form of intense light to tissue anywhere within the intracranial space in conjunction with real-time MR thermography, which is used to monitor the delivery of this energy producing a 5- to 20-mm-diameter ablation zone ^145^. MgLiTT has been used for the treatment of various epileptogenic conditions, including MTLE, hypothalamic hamartomas, periventricular nodular heterotopia, tuberous sclerosis, cortical dysplasia, cavernous hemangiomas, and insular encephalomalacia ^146, 147^. In MTLE, seizure outcome was similar to or slightly worse than that of open surgery while cognitive outcome was better ^107, 108, 145, 147– 150^. In a series of 71 hypothalamic hamartoma patients operated with MgLiTT, 93% were free of gelastic seizures at one year. One patient experienced a significant memory deficit, and one patient experienced worsening diabetes insipidus. Thus, MgLiTT represents a safe and effective surgical option for the treatment of hypothalamic hamartomas ^151^. Advantages of MgLiTT include its minimally invasiveness with better tolerability and quicker recovery time, fewer major complications, possibly a lower degree of adverse cognitive effects and the fact that it can be repeated and does not preclude subsequent surgery ^10, 147^.

Total or partial destruction of the epileptogenic zone as tailored to each patient by SEEG exploration is the goal of SEEG-guided RFTC. A radiofrequency generator connected to the electrode contacts is used to produce multiple SEEG-guided RFTC lesions of epileptic foci ^152^. SEEG-guided RFTC has been performed in hippocampal sclerosis, periventricular nodular heterotopias, focal cortical dysplasia, tuberous sclerosis and in patients with normal MRI ^152– 160^. According to a recent review, seizure-free rate was rather heterogeneous across studies with a pooled seizure-free rate of 23% and a pooled responder rate of 58% ^159^. The highest responder rate was observed in patients with nodular heterotopia, the lowest in patients with normal MRI. The pooled rate of permanent neurologic deficit was 2.5%. The authors concluded that SEEG-guided RFTC represents a safe treatment for patients with drug-resistant focal epilepsy when conventional resective surgery is not feasible ^159^.

In a significant number of patients with medically refractory epilepsy, curative epilepsy surgery cannot be offered since there are multiple epileptogenic zones, the epileptogenic zone cannot be localized at all, or the epileptogenic zone is located within functionally relevant brain areas. For these patients, various neurostimulation techniques, including vagus nerve stimulation (VNS), DBS, and RNS, are becoming an increasingly accepted treatment option and therefore should be considered in every patient with medically refractory epilepsy who is unsuitable for surgery ^161^. However, in contrast to resective and ablative epilepsy surgery, these neuromodulatory techniques represent palliative procedures resulting in seizure reduction at best, and seizure-free outcome only in exceptional cases. A recent review found low- to moderate-quality evidence for the efficacy and safety of VNS, DBS, and RNS ^161^. Head-to-head comparisons between different neuromodulatory techniques are missing and, owing to methodological difficulties, most probably will not be available in the near future ^161^. Therefore, at present, it is not possible to decide which treatment modality is best suited for a specific patient population and thereby allow personalized treatment decisions.

## Conclusions

Epilepsy surgery represents a valuable treatment options for 10 to 50% of patients with drug-resistant epilepsy. The most common histopathological findings in epilepsy surgery specimens are hippocampal sclerosis in adults and focal cortical dysplasia in children. Whereas presurgical evaluations and surgeries in patients with mesial temporal sclerosis and benign tumors recently decreased in most centers, non-lesional patients, patients requiring intracranial recordings, and neocortical resections increased. More evaluated patients did not undergo surgery since patients were not suitable for surgery (due mainly to missing identification of a circumscribed epileptogenic zone) or since more patients offered surgery by physicians opted against surgery.

The prerequisite for successful epilepsy surgery is a thorough presurgical evaluation clearly defining epileptogenic and essential brain areas and designing a resection plan in each individual patient. Phase 1 presurgical investigations include high-resolution MRI, video scalp EEG, and detailed neuropsychological assessment. If these investigations yield inconclusive or ambiguous results, additional non-invasive techniques can be used. If the epileptogenic zone cannot be localized with sufficient certainty with non-invasive techniques and the patient is still considered a reasonable surgical candidate, intracranial recordings with depth or subdural electrodes (or both) can be applied during a so-called phase 2 evaluation.

Recent developments in neurophysiological techniques (high-density EEG, MEG, ESI and MSI, EEG-fMRI, and recording of pHFOs), structural MRI (ultra-high-field imaging at 7 Tesla, novel imaging acquisition protocols, and advanced image analysis [post-processing] techniques), functional imaging (PET and SPECT co-registered to MRI), and fMRI significantly improved non-invasive presurgical evaluation and have opened the option of epilepsy surgery to patients previously not considered surgical candidates.

Technical improvements of resective surgery techniques facilitate successful and safe operations in highly delicate brain areas like the perisylvian area in operculoinsular epilepsy. Novel less-invasive surgical techniques include stereotactic radiosurgery, MgLiTT, and SEEG-guided RFTC.

## Abbreviations

3D, three-dimensional; AAN, American Academy of Neurology; CEEG, extraoperative intracranial electroencephalography through open craniotomy; CSF, cerebrospinal fluid; DBS, deep brain stimulation; DTI, diffusion tensor imaging; ECoG, intraoperative electrocorticography; EEG, electroencephalography; EEG-fMRI, electroencephalography-functional magnetic resonance imaging; ESI, electrical source imaging; ETLE, extratemporal lobe epilepsy; FDG, 18F-fluoro-deoxyglucose; FLAIR, fluid-attenuated inversion recovery; fMRI, functional magnetic resonance imaging; HD-EEG, high-resolution electroencephalography; IEEG, intracranial electroencephalography; MEG, magnetoencephalography; MgLiTT, magnetic resonance-guided laser interstitial thermal therapy; MR, magnetic resonance; MRI, magnetic resonance imaging; MSI, magnetic source imaging; MTLE, mesial temporal lobe epilepsy; PET, positron emission tomography; pHFO, pathological high-frequency oscillations; rCBF, regional cerebral blood flow; RNS, responsive neurostimulation; SEEG, stereotactic intracerebral electroencephalography; SEEG-guided RFTC, stereotactic intracerebral electroencephalography-guided radiofrequency thermocoagulation; SISCOM, subtraction single-photon emission computed tomography co-registered to magnetic resonance imaging; SPECT, single-photon emission computed tomography; STATISCOM, statistical ictal single-photon emission computed tomography co-registered to magnetic resonance imaging; SVR, support vector regression; TLE, temporal lobe epilepsy; VNS, vagus nerve stimulation
